# Violence against black women: healthcare professionals’ knowledge and practices

**DOI:** 10.1590/0034-7167-2024-0250

**Published:** 2025-10-03

**Authors:** Diully Siqueira Monteiro, Ivaneide Leal Ataíde Rodrigues, Erlon Gabriel Rego de Andrade, Laura Maria Vidal Nogueira, Ingrid Fabiane Santos da Silva, Aloma Sena Soares, Giovanna Paraense da Silva

**Affiliations:** I Universidade Federal do Pará. Belém, Pará, Brazil; II Universidade do Estado do Pará. Belém, Pará, Brazil; III Universidade Federal do Rio de Janeiro. Rio de Janeiro, Rio de Janeiro, Brazil.

**Keywords:** Intimate Partner Violence, Violence Against Women, Racism, Health Personnel, Public Health, Violencia de Pareja, Violencia contra la Mujer, Racismo, Personal de Salud, Salud Pública

## Abstract

**Objectives:**

to analyze Primary Healthcare and Health Surveillance professionals’ knowledge and practices in the face of violence against black women in the domestic context.

**Methods:**

a qualitative, descriptive study, carried out in a municipality in the Metropolitan Region of Belém/Pará/Brazil. Thirty-four healthcare professionals participated, interviewed individually, with semi-structured script. The *corpus* was subjected to lexical analysis with *Interface de R pour les Analyses Multidimensionnelles de Textes et de Questionnaires* 0.7/alpha 2 using Descending Hierarchical Classification.

**Results:**

of the 34 participants, 24/70.59% were women and 12/35.29% were between 40-49 years old. Ninety-four text segments were identified, using 77/81.91%, divided into six lexical classes. Classes were organized into two thematic axes, presenting knowledge and care/management practices on the topic.

**Final Considerations:**

knowledge was based on the recognition of the unequal power relationship between genders, and practices revealed limitations, which may reinforce the vulnerability of black women in situations of violence.

## RESUMEN

**Objetivos:**

analizar conocimientos y prácticas de los profesionales de la Atención Primaria de Salud y de Vigilancia de la Salud frente a la violencia contra las mujeres negras en el contexto doméstico.

**Métodos:**

estudio cualitativo, descriptivo, realizado en un municipio de la Región Metropolitana de Belém/Pará/Brasil. Participaron 34 profesionales de la salud, entrevistados individualmente, mediante un guión semiestructurado. El *corpus* fue sometido a análisis léxico con el *software Interface de R pour les Analyses Multidimensionnelles de Textes et de Questionnaires* 0.7/alpha 2. utilizando la Clasificación Jerárquica Descendente.

**Resultados:**

de los 34 participantes, 24/70,59% eran mujeres y 12/35,29% tenían entre 40-49 años. Se identificaron 94 segmentos textuales, aprovechando un 77/81,91%, divididos en seis clases léxicas. Las clases se organizaron en dos ejes temáticos, presentando conocimientos y prácticas de atención/gestión sobre el tema.

**Consideraciones Finales:**

el conocimiento se basó en el reconocimiento de la relación desigual de poder entre los géneros, y las prácticas revelaron limitaciones que podrían reforzar la vulnerabilidad de las mujeres negras en situaciones de violencia.

## INTRODUCTION

Violence is considered a violation of human rights and a major public health problem due to its ability to produce deleterious effects on the health-disease process. Recognized as actions that cause harm and undermine subjects’ integrity, both individually and collectively, violence involves power relations over others, resulting in physical, psychological and social harm^(1-4)^.

When dealing with domestic violence against women, diverse forms of violence and many factors related to perpetrators are identified, as perpetrators can be multiple and have different degrees of social ties with victims, and, recurrently, the main person identified is the intimate partner^(5)^.

This type of violence is permeated by patriarchy, a phenomenon marked by a socially constructed and shared power structure, whose center is occupied by the male figure, generating repercussions in women’s lives, whether at the individual, relational, cultural or environmental levels. This phenomenon favors the maintenance of structures of domination and exploitation of women, increasing social vulnerability^(6,7)^.

For black women, oppression incorporates racism, historically related to the process of denial of social rights. In Brazil, the black population has experienced continuous processes of social exclusion, originating from the period of slavery, which created conditions of subordination in relation to wealthy groups. Given this historical burden, black women suffer social repercussions derived from racism, including patriarchy and other discriminatory systems, determining or strengthening conditions of greater vulnerability^(8,9)^.

In this context, the Atlas of Violence, published in 2023 by the Institute of Applied Economic Research and the Brazilian Public Security Forum, highlights the increase in violence and its lethality in Brazil. This document pointed out that, from 2011 to 2021, the rate of femicide in the domestic environment increased by 4.72%. In 2021, black women were almost twice as likely to die as non-black women, with a national rate of approximately 4.3 black women killed for every 100,000 inhabitants, demonstrating the persistence of the problem^(10)^.

It is worth noting that, from 2011 to 2021, the North region had the highest rates of femicide among black women, with an increase of 2.9% in Pará. Also in 2020 and 2021, during the COVID-19 pandemic, the state showed an increase of 12.7%, above the national value (0.5%), recorded in the same period^(10)^.

The overview presented shows the lethality of violence against women in the North region, especially in Pará. Although there are legal and health frameworks to protect this population, which involve careful data collection on the phenomenon, the possibility of underreporting is admitted^(11)^. In this context, Primary Healthcare (PHC), as the gateway to the Brazilian Health System (In Portuguese, *Sistema Único de Saúde* - SUS), plays a fundamental role in recognizing situations of violence and preventing lethality, with qualified listening and compulsory reporting as essential tools^(12-14)^.

Thus, it is understood that it is relevant to develop studies on the topic, due to the possibility of encouraging critical-reflective attitudes in healthcare professionals’ daily lives and broadening the view on the challenges and strengths inherent in the recognition and prevention/reduction of this social phenomenon in the health field.

## OBJECTIVES

To analyze Primary Healthcare and Health Surveillance professionals’ knowledge and practices in the face of violence against black women in the domestic context.

## METHODS

### Ethical aspects

The study complied with Resolution 466/2012 of the Brazilian National Health Council, with approval from the Research Ethics Committee of the Nursing Undergraduate Course at the *Universidade do Estado do Pará.* Institutional authorization was obtained from the Health Department of the municipality where the study was conducted. Participants’ acceptance was obtained through signing the Informed Consent Form (ICF). To ensure identity confidentiality, alphanumeric codes were used, with the letters CP and TV, in reference to the respective terms “care professional” and “surveillance technician”, and the numerical sequence of the order of interviews.

### Study design

This is a descriptive study, with a qualitative approach, guided by the COnsolidated criteria for REporting Qualitative research (COREQ) checklist^(15,16)^.

### Study settings and data source

It was carried out in the Basic Health Units (BHU) and in the Health Surveillance Department of a municipality that makes up the Metropolitan Region of Belém, capital of the state of Pará, Brazil. During the study, the municipal care network consisted of 17 BHU, 25 family health teams, nine oral health teams and four Expanded Family Health Centers (In Portuguese, *Núcleos Ampliados de Saúde da Família* - NASF).

Thirty-four professionals participated, representing 40.48% of the total of 84 higher education healthcare professionals who worked in Family Health Strategy, NASF and Health Surveillance teams of the municipality. The inclusion criterion was defined as professional experience of at least one year in PHC and/or Health Surveillance. It was decided to exclude those who were away from their activities during the data production period, regardless of the cause.

However, there were no exclusions, refusals or withdrawals, which is why the number of participants was defined by theoretical saturation, a criterion used to end data production, when it was identified that the set of statements was sufficient to understand the object of study^(17)^.

### Data collection and organization

Participants were selected based on convenience and approached personally, individually, at the locations where they worked. Those who accepted were taken to a previously reserved room with service managers, ensuring privacy and an environment conducive to dialogue, with 70% alcohol gel and facilities to increase health safety in the context of the COVID-19 pandemic. At this time, the research project and the ICF were presented to participants for clarification and signature, formally declaring their acceptance.

Data production took place from May to August 2021, through individual interviews, conducted only by the first author, a nurse and student of the Multiprofessional Residency Program in Family Health Strategy at a public university based in Belém. During the interview, only the researcher and the participant were in the room, and she was previously trained in orientation meetings for research activities, making up the theoretical workload of the residency course.

Considering that PHC services and the Health Surveillance Department of the municipality were internship sites for the researcher during the course, she naturally established a prior relationship with some of the professionals who worked there. In view of this, all participants knew the trajectory and certain academic and professional aspirations of the researcher as well as the fact that this study was a mandatory requirement for completing the course.

In order to guide the interviews, a semi-structured script was used, prepared by the research team and consisting of two sections. With seven questions about the sociodemographic and professional profile, the first section allowed recording participants’ work setting, age, sex, professional category, qualification, length of service and participation in continuing education spaces. With nine subjective questions, the second made it possible to explore the object of study, focusing on knowledge about violence against black women and care and management practices in this situation, which were unfolded in the dialogue with participants.

No pilot test or validation study was conducted with this instrument, nor was a field diary or other complementary technique used to produce data, since the interviews proved sufficient to capture the object. Considering data completeness and clarity, it was not necessary to repeat interviews, and it was also decided not to share the transcripts with participants so that they could check and endorse them, in order not to compromise the spontaneity of statements. After consent, the interviews were audio-recorded in MP3 format with an electronic device, and lasted, on average, 40 minutes.

### Data analysis

The interviews were transcribed and compiled into a *corpus*, meeting the requirements of the lexical analysis software *Interface de R pour les Analyses Multidimensionnelles de Textes et de Questionnaires* (IRaMuTeQ^®^) version 0.7, alpha 2, for processing. IRaMuTeQ^®^ uses functionalities of the R software, enabling statistical and textual analysis of the *corpus*, with reliability and technical-scientific rigor^(18)^.

During processing, the software generated a dendrogram, a graphical representation of Descending Hierarchical Classification (DHC), one of the analytical modalities of IRaMuTeQ^®^. With DHC, the *corpus* was divided into lexical classes, consisting of words organized vertically, according to their chi-square (X^2^), statistical values that demonstrate the associative strength of words to form classes. The text segments (TS) corresponding to each word were read carefully to understand and interrelate their meanings, allowing the grouping of classes into thematic axes^(19,20)^.

It is important to note that, when processing DHC, IRaMuTeQ^®^ identifies the best possibility for forming classes according to the statistical and semantic factors of the *corpus*. This often determines the use of a portion of the TS, considering 75% as the minimum percentage for the analysis to be reliable^(19,20)^.

Thus, two axes were derived from subjective data analysis, each consisting of three classes, according to their complementarities and similarities, which were interpreted and discussed based on the relevant scientific literature on the subject and on official legal and health documents. In turn, the sociodemographic and professional profile data were organized in a Microsoft Office Excel^®^ version 2016 spreadsheet and analyzed with descriptive statistics to obtain the absolute values and respective percentages. The data analysis and interpretation processes were conducted by the first five authors.

In order to preserve its originality, the study report was not sent to participants, which is why they did not provide opinions on the results, but the intention is to share the article as soon as it is published.

## RESULTS

Of the 34 participants, 28 (82.35%) worked in healthcare activities in PHC, and six (17.65%) worked as technicians in the Health Surveillance Department, 24 (70.59%) of whom were female. Ages ranged from 29 to 56 years, with an average of 35 and a predominance of the 40 to 49 age group (n=12; 35.29%). Concerning professional category, 24 (70.59%) were nurses, five (14.71%) were physicians, three (8.82%) were physiotherapists, and two (5.88%) were occupational therapists. Regarding qualifications, 25 (73.53%) declared having a *lato sensu* and/or *stricto sensu* graduate degree.

The length of professional experience was distributed as follows: 15 (44.12%) reported six to ten years, followed by eight (23.53%) with one to five years; seven (20.59%) with 11 to 15 years; three (8.82%) with 21 years or more; and one (2.94%) with 16 to 20 years. Regarding continuing education, 31 (91.18%) reported never having participated in training spaces on violence against women.

The *corpus* consisted of 34 texts, corresponding to the set of interviews. Ninety-four TS were identified, with 77 being used, making up 81.91% of the *corpus*, which is why this study revealed the main subjective data and allowed us to understand the object of study, dispensing with secondary data, which constitute the unused percentage of the *corpus* (18.09%).

**Figure 1:**
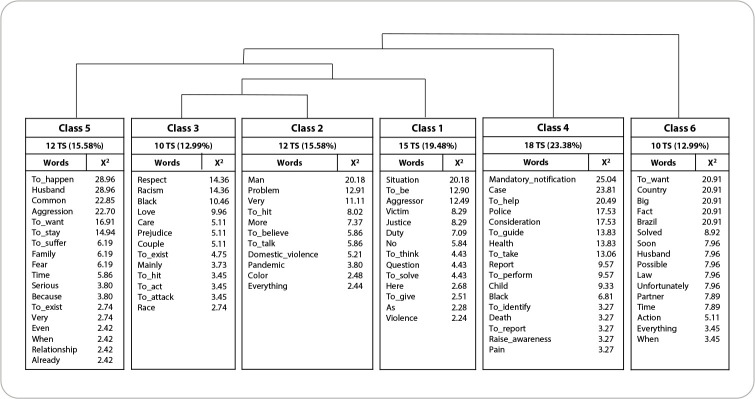
Dendrogram of Descending Hierarchical Classification, Belém, Pará, Brazil, 2021

A total of 3,228 occurrences (forms or words) emerged, of which 1,048 were distinct words and 678 had a single occurrence (hapax). Using DHC, the *corpus* was subdivided into three *subcorpora* and six lexical classes. Following the order of partition, the first *subcorpus* consisted of class 6, the second, class 4, and the third, classes 5, 1, 2, and 3 (Figure 1).

Composed of 10 TS (12.99% of the *corpus*), class 6 presents 16 representative words, considering the X^2^ values. In turn, class 4 presents 18 TS (23.38%) and 17 representative words, being the largest; class 5, 12 TS (15.58%) and 18 representative words; class 1, 15 TS (19.48%) and 14 representative words; class 2, 12 TS (15.58%) and 11 representative words; and class 3, 10 TS (12.99%) and 13 representative words. Thus, together, classes 6 and 3 constitute the smallest (Figure 1).

Organizing the classes into two thematic axes, the first axis grouped classes 6, 3 and 2, and the second, classes 4, 5 and 1. These axes were named according to the content of the classes, and are found below, highlighting representative words, meanings and some emblematic excerpts.

### Thematic axis 1 - Healthcare professionals’ knowledge about domestic violence against black women (classes 6, 3 and 2)

This axis presents professionals’ understanding of domestic violence, explaining that the recognition of violence occurs both through the set of subtle acts and through harmful actions, since all of them constitute violent and domineering practices against black women. This understanding encompasses aspects of public safety, which are understood as fragile in combating violence and its possible repercussions on victimized women’ lives.

Words in class 6, such as “to want” (X^2^=20.91), “husband” (X^2^=7.96) and “partner” (X^2^=7.89), reveal that professionals have already detected some forms of violence, such as sexual violence, with their partners exercising domination over women in non-consensual sexual intercourse. Moreover, other violent practices cause fear and dependence, whether emotional, financial and/or psychological. In professionals’ perception, these practices result in harm to women:

*The partner or husband believes he has the right to order the woman around, and she has the duty to obey, without having a choice. Many women say, “Sometimes I don’t want to have sex, but I feel very submissive”. They are dependent on their husbands and don’t go after what they really want.* (ST 11)

*A husband who abuses his wife creates situations of dependency, and out of fear, insecurity, fear of being alone, not having the means to support herself, among other situations,* [she] *ends up staying* [with her husband]. (ST 14)

As for black women, some words from class 3, such as “racism” (X^2^=14.36) and “prejudice” (X^2^=5.11), show the recognition that, in their case, violence is even more serious, as it brings the racist component, which constitutes a strong legacy in the reproduction of collective thought over time:

*Violence against women involves beatings, humiliation,* [psychological] *manipulation, everything that hurts. But prejudice based on color is cruel. Unfortunately, racism exists.* (TV 1)

*Black women suffer much more. Being black means carrying a burden.* (ST 5)

In class 2, it is worth highlighting that the word “pandemic” (X^2^=3.80) reflects the context of the health crisis caused by COVID-19 and its repercussions in the social and health spheres, with an increase in the number of cases of domestic violence due to forced/imposed coexistence due to health measures, especially during lockdown periods:

*Violence has increased significantly during this pandemic. We are seeing many cases of femicide in the news, especially men killing their wives and girlfriends* [...]. (TV 6)

*The silence has become stronger during the pandemic* [...], *there can be no contact to talk* [due to restrictions to control COVID-19]. *Women remain in violent relationships for many reasons: fear, shame, hope that their partner’s behavior will change, economic and psychological dependence, and social isolation*. (ST 13)

In this context, the words “respect” (X^2^=14.36) and “love” (X^2^=9.96), which appear in class 3, demonstrate that professionals understand violence, including against black women, as a phenomenon motivated, above all, by the dynamics of the interpersonal relationship between victim and perpetrator. This portrays social values and norms that can influence comprehensive care for victims:

*Many femicides, especially men killing their wives or girlfriends,* [occur] *because there is no longer respect and love. This business of color, for me, is not important. Now everyone says “black lives matter”, but we have to say that all lives matter, especially these couples* [who live in conflict], *to reestablish love and reduce hate.* (TV 6)

*Unconsciously, we all judge someone, even if we don’t want to. I have a great affection for my patients. There were times when I silently judged them, and I understand everything they went through, but I think that allowing a lack of respect in a relationship leads to violence. Love is necessary in a healthy relationship.* (ST 28)

Professionals strongly point out that, in many contexts, the social structure is still centered on the male figure as the holder and influencer of romantic relationships, in the same way that social thinking, when anchored in racism, drives the phenomenon of violence:

*We still come across individuals who believe in the superiority of human beings based on the color of their skin and women as objects. We see many cases of men who do not accept separation, beat* [their wives], *break things, threaten and kill, and there are even those who hide their bodies*. (TV 4)

*Domestic violence certainly hurts black women more, since they are the most vulnerable, if we take into account factors such as unemployment and emotional dependence. In this family, it is usually the man who works* [...]. (ST 5)

This sociocultural dimension, presented by the words “man” (X^2^=20.18) and “very” (X^2^=11.11), in class 2, exposes the patriarchal logic and capitalist influence that, together, determine stereotypes for men and women in social imagination, and the dynamics of use of power in domestic violence. It is noteworthy that, when discussing the phenomenon, professionals sometimes generalize the female context, shadowing aspects that constitute black women’s social universe, but which need to be recognized in their singularities:

*Men who are perpetrators by nature will question the woman’s clothing, her behavior, the time she was out; they will minimize her story and question her word. Society is based on this: men have the power! Until recently, they were the ones who dominated the* [job] *market. But women have to be careful and always circumvent this power.* (ST 2)

*Historically, men think they own women. The culture is still very much that men are the ones who own everything, especially in* [romantic] *relationships.* (ST 4)

On the other hand, the words “Brazil” (X^2^=20.91), “law” (X^2^=7.96) and “unfortunately” (X^2^=7.96), in class 6, characterize domestic violence, especially in the daily lives of black women, as a social problem of national proportions, with a high degree of injustice. Participants expressed weaknesses that occur in the timely and effective application of protective measures, provided for by law, resulting in conditions of impunity, to the extent that victims, after resorting to the authorities constituted for this purpose, will often still be subject to the risk of suffering other episodes of violence, including lethal violence:

*Brazil is an extremely unequal country, with archaic criminal legislation,* [which] *results in this* [domestic violence]. *She* [the woman] *will report her partner, but he will not be arrested. When she dies,* [the authorities] *will look into the report.* (ST 15)

*Crime and impunity, because of laws* [that are not enforced], *are a big problem, at least in Brazil, and I believe that this applies to everyone, men and women.* (ST 18)

In this context, it is also worth highlighting the perception that female passivity favors violence as a “mitigating factor”, since the fact that women do not react constitutes a type of acceptance. Thus, the phenomenon is reduced to individualized expressions about the relationship between victims and perpetrators. However, this representation of the collective, which attempts to explain the cycle of domestic violence, does not consider the phenomenon social markers:

[...] *most cases of domestic violence against women come from problems with alcohol, drugs or mental illness in the husband.* (TV 2)

*Violence is attacking someone in any way. The fact that it occurs in a loving relationship, with the presence of feelings,* [causes] *the intense love between the couple to reduce the pain of the moment, and a vicious cycle of abuse is born. The man changes his attitude after the violence and offers a pleasant coexistence, which eases the situation. The woman is reassured by her husband’s romantic attitudes. We noticed that the problem was quickly resolved. If the situation were so serious, the woman would immediately leave the perpetrator/partner.* (ST 3)

### Thematic axis 2 - Assistance and management practices for black women in situations of domestic violence (classes 4, 5 and 1)

This axis makes it possible to understand professionals’ actions when dealing with black women in situations of domestic violence within the scope of PHC.

The words identified in class 4, such as “Mandatory_notification” (X^2^=25.04), “report” (X^2^=9.57) and “to perform” (X^2^=9.57), point to the challenges of reporting interpersonal violence events in PHC. The words are associated with the idea of notification as an element of criminal evidence for civil justice, even though the registration of the notification form serves only for epidemiological purposes, legally protecting notifying professionals:

*I believe in mandatory reporting for more than just epidemiological purposes. If it happens here, I will identify it in the report, include it in medical records and provide advice.* (ST 5)

*Many women believe that they can endure the aggression and continue in the relationship as a way of protecting their children. Mandatory notification and any other type of notification are not performed here, taking these situations into account. This is how the healthcare professional will subject themselves to contradiction, leading to a decrease in their professional credibility.* (ST 26)

Professionals’ understanding of compulsory notification as a type of civil complaint explicitly points to the insecurity in carrying it out, as they consider domestic violence as a cycle that will not be easily interrupted, especially when there is some type of dependence of victims in relation to perpetrators.

Thus, words such as “case” (X^2^=23.81) and “police” (X^2^=17.53), together with words from class 5, such as “to happen” (X^2^=28.96) and “aggression” (X^2^=22.70), present the context of denial of the health sector’s attributions in the face of violence against black women, highlighting the portrait of institutional racism in public health, which often occurs due to the fear that professionals express when faced with the possibility of also finding themselves in a vulnerable situation:

*Violence is a police matter. There is no reason for us to be here, in the health unit, discussing this issue.* (ST 9)

[...] *I think that, depending on the severity* [of the case], *I will not provide care. I am afraid, because it may happen that the woman returns* [reconnects] *with her husband after the assault, and I, as a nurse, will be scarred by him* [...], *the pain caused by violence should be reported to the police.* (ST 20)

Thus, they considered that violence should be addressed only within the scope of public safety, as evidenced by professionals’ insecurity to carry out care actions for victims, added to the fear of retaliation by perpetrators against professionals in the workplace itself and/or in other environments, including the possibility of becoming lethal victims of violence. This portrait of denial and the pact of silence in public health, especially in PHC, in a pandemic context, possibly intensified a bleak scenario for femicide.

When identifying situations of domestic violence, depending on the characteristics and intensity of injuries, they advised the victims, referring them to other levels of healthcare. In view of this, in class 4, words such as “to help” (X^2^=20.49) and “to guide” (X^2^=13.83) are associated with problem-solving behaviors when faced with a black woman in a situation of violence. However, the practices mentioned indicate that professionals reduced them to care for minor physical aggressions, evidencing limitations in expanded care. They recognized that the participation of a multidisciplinary team is necessary to support care actions, indicating professionals who can compose it, such as psychologists and social workers:

*I also think that what can help strengthen are professionals such as psychologists and social workers, who* [help reduce] *the pain of violence.* (ST 20)

[...] *today, in the* [health] *unit, if a situation occurs, with clear signs of aggression, violence itself against black women, I will only provide healthcare, which will not extend to social care.* (ST 24)

Finally, the words “perpetrator” (X^2^=12.49) and “violence” (X^2^=2.24), in class 1, together with the words “to suffer” (X^2^=6.19), “family” (X^2^=6.19) and “relationship” (X^2^=2.42), in class 5, engender recognition of the repercussions of violence on family members. In the intra-family context, domestic violence is seen as an aggravating factor for black women, with harmful effects on their children. Understanding that this phenomenon represents an indirect attack on offspring, professionals stated that situations of violence imply social and health damages for children as well, demanding other care actions:

*When aggression occurs against women, it is harmful in itself,* [because] *it causes damage to the entire family structure, especially when there are children. In this case,* [it] *is also violence against children.* (ST 1)

*Violence causes pain to women, and everyone around them suffers.* (ST 21)

It was found that professionals strongly explored the idea of dependence of victims who have children, as they were seen as those who most endure situations of aggression, in order to protect them and prevent them from suffering deprivation and/or psychological/emotional shocks.

## DISCUSSION

The data highlighted professionals’ knowledge about domestic violence against black women and a set of practices in response to this reality. It is known that this violence encompasses the complex intersection of social oppressions, an expanded context that can be didactically understood as an intersection of the microsocial and macrosocial spheres. The microsocial sphere corresponds to the singular representations and actions of subjects, differing from the macrosocial sphere, indicated by the structural implications of historical, political and social extension, which involve society and, consequently, have repercussions on women^(21)^.

In the context of violence, recognition of the microsocial sphere is marked by power relations between men and women, rooted in the patriarchal system, in which domination in gender relations influences daily practices and is based on the rigid definition of social roles. These forms of domination and exploitation are reciprocally related in a structure composed of class, gender and race^(22-24)^.

Expressions of violence against black women find convergences and possibilities of interpretation in light of theoretical frameworks available in scientific literature, such as the theory of intersectionality, through which it is analyzed how certain social and cultural categories intertwine, covering relationships between various elements, highlighting ethnicity and sexuality, but also class, gender and race, in a multidimensional perspective^(21)^.

This consideration is made considering that this theory allows us to reflect on the fact that many forms of oppression and their corresponding inequities are firmly intertwined and mutually reinforcing, creating unique experiences of discrimination. This tends to result in greater vulnerability of women to violence, both in the domestic environment and in other social spaces, causing or exacerbating phenomena of marginalization, especially among black women^(21)^.

Thus, the results of this research are in line with a study carried out in Marabá (PA), which analyzed healthcare professionals’ perceptions on the subject, indicating the domestic environment as the epicenter of violence against women, as well as the close domestic bond with perpetrators and the fear of breaking the situation of violence as its main influencers^(25)^. In the same study, it was found that, at times, disrespect towards women goes beyond the home environment and reaches the legal environment, characterized, however, by obstacles to protecting women and by the ineffective judgment of perpetrators^(25)^.

Although the results of this research point to violence against black women committed by intimate partners in the domestic environment, violence extends to a plurality of perpetrators and can be shared by family groups and other social groups. Furthermore, it can encompass different patterns of interaction, based on racism and/or sexism. This diversity must be considered to broaden the scope of coping by recognizing existing social inequalities^(26)^.

As a violation of human rights, domestic violence has direct health effects and can be lethal to victims. In this regard, participants recognized violence beyond its physical aspects, including its psychosocial repercussions. However, they expressed insecurity regarding the actions they should take, exposing the need to improve care/management skills and qualify public security so that they have the necessary security to welcome victims, solve the condition of violence and/or mitigate its biopsychosocial repercussions^(25)^. This can occur especially in PHC, where close relationships between professionals and human groups make it possible to address different problems^(27-29)^.

Archaic and mistaken popular conceptions, such as the idea that women “like to be beaten”, deny the complexity of the problem so that the persistence of victims with perpetrators is trivialized and not seen as the result of multiple factors, attributing to violence an individual character arising from supposedly specific aspects of the female personality, such as the level of increased tolerance and/or the hope that perpetrators will change. These conceptions result in the denial of the cycle of violence and the silencing of victims^(27)^.

Therefore, it is essential to analyze social factors jointly by class, gender and race. Based on intersectionality, this analysis makes it possible to understand the ways in which racism, patriarchy, class oppression and other discriminatory systems operate, which create inequalities and structure/institutionalize subordination and vulnerabilities between people^(23,30)^.

Given the multiplicity of the female gender, the union of the phenomena of racism and sexism is identified, elements that produce violent effects on women, especially black women^(31)^. This oppressive logic was reproduced in the findings of this study, when they pointed out certain knowledge of professionals, anchored in the relativization of suffering caused by violence, associating it with affective and intimate aspects as a result of the expression of social subjectivity of racism and sexism, of which, often, not even they are aware.

Actions to combat violence have been greatly impacted by the COVID-19 pandemic, which has changed access to PHC services to reduce the spread of the virus, as well as the relational dynamics of families due to the need for their members to remain isolated at home for days or weeks, increasing the number of cases of violence against women. In this scenario, evidence indicates that women were the most affected by violence during and after confinement^(32-34)^.

Situations characterized by increased stress due to school closures and high unemployment rates, in addition to the need for some women to remain confined in the same home as their perpetrators, combined with financial dependence on them, among other factors aggravated by the pandemic, have worsened the scenario of domestic violence. In addition to this, underreporting of violent events and social isolation of victims, resulting from the pandemic and the lack or absence of support networks, have contributed to strengthening domestic violence^(34)^.

In this context, the daily challenges of the violence care network were permeated by factors such as the reinvention of ways of creating and recreating links with the community, with budgetary restrictions in PHC and the incorporation of neoliberal regulations that, in turn, resulted in the prioritization of care for acute demands, of biological origin, rather than comprehensive and longitudinal care, with a community and family approach^(35)^.

Therefore, from the perspective of intersectionality, it is understood that it is necessary to recognize violence against women as a silent and invisible pandemic, constructed as a social phenomenon and continuously processed in the shadows of relationships between human groups, as occurred in the midst of COVID-19^(21)^. This highlights the need to promote intersectoral actions aimed at the female public, especially black women, in order to guarantee links with services, protection and comprehensive care, considering their particularities^(34)^.

Difficulties in comprehensive care and the impasses of compulsory notification, mentioned by professionals, demand the improvement of the technical-operational structure of PHC, with the implementation of protocols and service flows for prevention activities, reception/care, notification and follow-up of cases as well as for activities to promote the culture of peace and epidemiological monitoring of domestic violence^(36,37)^.

It is important to emphasize the importance of strengthening the articulation of these activities with other levels of healthcare, in accordance with instructions of Organic Health Law 8,080/1990^(38)^, incorporating the area of public safety, as guided by Law 14,550/2023, which deals with the implementation of urgent protective measures for victims and their dependents^(39)^.

In relation to legal support, Laws 10,778/2003 and 13,931/2019 deal with the identification and mandatory reporting of suspected and confirmed cases of violence against women. They jointly define the measures that must be taken in the public and private sectors, highlighting, in the health field, reception and reporting as objects of identification for appropriate measures and statistical purposes. In addition, they indicate the obligation to report the occurrence to the police authority within the first 24 hours^(40,41)^.

Faced with the need to report violence, there is the difficulty of handling complex situations that involve it, such as the fear of becoming vulnerable when reporting and filing a police report, exposing ideas that refer to the need to delegate responsibility for carrying out this procedure to other sectors, such as sectors in the civil/criminal area^(36,37)^.

The challenges of reporting and dealing with cases of domestic violence may be related to the lack or shortage of qualified training^(42)^, identifying feelings of helplessness and unpreparedness in the face of situations experienced in practice, which reveal flaws in the care provided to victims. Therefore, it is essential to promote ongoing education activities in order to ensure good care for victimized women, based on equity, comprehensiveness and universality, which are the doctrinal principles of SUS^(43)^.

### Study limitations

As a limitation, it is worth noting that the study was conducted in a municipality in Pará, but it is understood that this aspect is representative, due to the important indicators of violence in this scenario. In order to broaden the scope of analysis, it is necessary to incorporate other municipalities, with representation from the state’s health regions, making it possible to articulate the care network for black women in situations of violence both in health and in other institutional sectors.

Moreover, the statements about the complex reality involving violence were impacted by the COVID-19 pandemic, which interfered with the availability and dimensioning of human resources as well as the dynamics of PHC and Health Surveillance services.

### Contributions to nursing, health or public policy

It is understood that the study contributes to the critical analysis of factors that have an impact on tackling domestic violence, especially in black women’s daily lives, serving as a theoretical contribution to help both public authorities to discuss, implement and assess measures that solve this problem or minimize its repercussions, and healthcare professionals to reflect on the topic and rethink their actions in the face of it.

## FINAL CONSIDERATIONS

It was evident that professionals’ knowledge about violence against black women was based on the recognition of the unequal power relationship between genders, characterized by conditions of subordination and vulnerability. In their experiences, domestic violence practiced by the spouse as the main perpetrator was identified as the predominant situation.

However, limitations were found in coping strategies that may contribute to increasing victims’ vulnerability. These limitations must be recognized to ensure good care in healthcare services, based on equity, comprehensiveness and universality.

Therefore, it is essential that PHC and Health Surveillance’ activities be provided with adequate technical and operational structure and tools, bringing them closer to other strategic sectors, such as public security, in order to establish comprehensive care for women who experience this serious problem.

## Data Availability

The research data are available within the article.
