# Supplementary material for: Violence against black women: healthcare professionals’ knowledge and practices
Source: Rev Bras Enferm. 2025 Oct 3;78(Suppl 2):e20240250. doi: 10.1590/0034-7167-2024-0250 (PMC12500077; doi:10.1590/0034-7167-2024-0250)
Supplement: Translation

# Translation: Violência contra mulheres negras: saberes e práticas de profissionais de saúde

**DOI:** 10.1590/0034-7167-2024-0250pt

**Authors:** Diully Siqueira Monteiro, Ivaneide Leal Ataíde Rodrigues, Erlon Gabriel Rego de Andrade, Laura Maria Vidal Nogueira, Ingrid Fabiane Santos da Silva, Aloma Sena Soares, Giovanna Paraense da Silva

**Affiliations:** I Universidade Federal do Pará. Belém, Pará, Brasil; II Universidade do Estado do Pará. Belém, Pará, Brasil; III Universidade Federal do Rio de Janeiro. Rio de Janeiro, Rio de Janeiro, Brasil.

**Keywords:** Violência por Parceiro Íntimo, Violência Contra a Mulher, Racismo, Pessoal de Saúde, Saúde Pública

## Abstract

**Objetivos:**

analisar saberes e práticas de profissionais da Atenção Primária à Saúde e da Vigilância em Saúde diante da situação de violência contra mulheres negras no contexto doméstico.

**Métodos:**

estudo qualitativo, descritivo, realizado em município da Região Metropolitana de Belém/Pará/Brasil. Participaram 34 profissionais de saúde, entrevistados individualmente, com roteiro semiestruturado. Submeteu-se o *corpus* à análise lexical com o *software Interface de R pour les Analyses Multidimensionnelles de Textes et de Questionnaires* 0.7/*alpha* 2, utilizando a Classificação Hierárquica Descendente.

**Resultados:**

dos 34 participantes, 24/70,59% eram mulheres e 12/35,29% tinham entre 40-49 anos. Foram identificados 94 segmentos de texto, aproveitando-se 77/81,91%, desdobrados em seis classes lexicais. As classes foram organizadas em dois eixos temáticos, apresentando saberes e práticas assistenciais/gerenciais acerca do tema.

**Considerações Finais:**

os saberes pautaram-se no reconhecimento da relação desigual de poder entre gêneros, e as práticas revelaram limitações, podendo reforçar a vulnerabilidade das mulheres negras em situação de violência.

## INTRODUÇÃO

A violência é considerada violação dos direitos humanos e um importante problema de saúde pública, devido à sua capacidade de produzir efeitos deletérios no processo de saúde-doença. Reconhecida por ações que causam danos e prejudicam a integridade do sujeito, tanto no caráter individual quanto no coletivo, a violência envolve relações de poder sobre o outro, resultando em danos físicos, psicológicos e sociais^(1-4)^.

Ao tratar da violência doméstica contra mulheres, identificam-se formas diversificadas de violência e muitos fatores relacionados ao agressor, pois os autores podem ser múltiplos e apresentar diferentes graus de vínculo social com as vítimas, sendo que, de modo recorrente, o principal identificado é o parceiro íntimo^(5)^.

Esse tipo de violência é atravessado pelo patriarcalismo, fenômeno marcado por uma estrutura de poder socialmente construída e partilhada, cujo centro é ocupado pela figura masculina, gerando repercussões na vida da mulher, seja no âmbito individual, relacional, cultural ou ambiental. Esse fenômeno favorece a manutenção das estruturas de dominação e exploração das mulheres, potencializando a vulnerabilidade social^(6,7)^.

Para a mulher negra, a opressão incorpora o racismo, historicamente relacionado ao processo de negação dos direitos sociais. No Brasil, a população negra vivenciou processos contínuos de exclusão social, oriundos do período de escravidão, que geraram condições de subalternidade em relação aos grupos abastados. Diante dessa carga histórica, a mulher negra sofre repercussões sociais derivadas do racismo, incluindo o patriarcalismo e outros sistemas discriminatórios, determinando ou fortalecendo condições de maior vulnerabilidade^(8,9)^.

Nesse contexto, o Atlas da Violência, publicado em 2023 pelo Instituto de Pesquisa Econômica Aplicada e pelo Fórum Brasileiro de Segurança Pública, evidencia o aumento da violência e sua letalidade no Brasil. Esse documento apontou que, no período de 2011 a 2021, a taxa de feminicídio no ambiente doméstico aumentou 4,72%. Em 2021, a mulher negra apresentava quase duas vezes mais risco de morrer que a não negra, com uma taxa nacional de, aproximadamente, 4,3 mulheres negras mortas para cada 100 mil habitantes, demonstrando a persistência do problema^(10)^.

Destaca-se que, nos anos de 2011 a 2021, a região Norte concentrou as maiores taxas de feminicídio entre mulheres negras, sendo que, no Pará, houve aumento de 2,9%. Também nos anos de 2020 e 2021, durante a pandemia de COVID-19, o estado apresentou aumento de 12,7%, acima do valor nacional (0,5%), registrado no mesmo período^(10)^.

O panorama apresentado mostra a letalidade da violência contra mulheres na região Norte, sobretudo no Pará. Embora existam arcabouços jurídicos e da saúde para proteger esse público, os quais perpassam por criterioso levantamento de dados acerca do fenômeno, admite-se a possibilidade de haver subnotificações^(11)^. Diante dessa conjuntura, a Atenção Primária à Saúde (APS), como porta de entrada do Sistema Único de Saúde (SUS), desempenha papel fundamental no reconhecimento das situações de violência e na prevenção da letalidade, tendo a escuta qualificada e a notificação compulsória como ferramentas essenciais^(12-14)^.

Assim, entende-se que é relevante desenvolver estudos sobre o tema, devido à possibilidade de estimular atitudes crítico-reflexivas no cotidiano dos profissionais de saúde e ampliar o olhar sobre os desafios e fortalezas inerentes ao reconhecimento e à prevenção/redução desse fenômeno social no campo da saúde.

## OBJETIVOS

Analisar saberes e práticas de profissionais da Atenção Primária à Saúde e da Vigilância em Saúde diante da situação de violência contra mulheres negras no contexto doméstico.

## MÉTODOS

### Aspectos éticos

O estudo obedeceu à Resolução nº 466/2012 do Conselho Nacional de Saúde, com aprovação no Comitê de Ética em Pesquisa do Curso de Graduação em Enfermagem da Universidade do Estado do Pará. E obteve-se autorização institucional da Secretaria de Saúde do município onde foi realizado o estudo. Obteve-se o aceite dos participantes por meio de assinatura do Termo de Consentimento Livre e Esclarecido (TCLE). Para garantir o sigilo da identidade, utilizaram-se códigos alfanuméricos, com as letras PA e TV, em alusão aos respectivos termos “profissional da assistência” e “técnico da vigilância”, e a sequência numérica da ordem das entrevistas.

### Tipo de estudo

Estudo descritivo, com abordagem qualitativa, orientado pelo *checklist COnsolidated criteria for REporting Qualitative research* (COREQ)^(15,16)^.

### Cenários do estudo e fonte de dados

Foi realizado nas Unidades Básicas de Saúde (UBS) e no Departamento de Vigilância em Saúde de um município que compõe a Região Metropolitana de Belém, capital do estado do Pará, Brasil. Durante o estudo, a rede de atenção municipal era constituída por 17 UBS, 25 equipes de saúde da família, nove equipes de saúde bucal e quatro Núcleos Ampliados de Saúde da Família (NASF).

Participaram 34 profissionais, representando 40,48% do total de 84 profissionais de saúde de nível superior que atuavam nas equipes da Estratégia Saúde da Família, dos NASF e da Vigilância em Saúde do município. Como critério de inclusão, definiu-se a experiência profissional de, no mínimo, um ano na APS e/ou na Vigilância em Saúde. Optou-se por excluir aqueles que estivessem afastados de suas atividades no período de produção dos dados, independentemente da causa.

Todavia, não houve exclusões, recusas ou desistências, motivo pelo qual o número de participantes foi definido por saturação teórica, critério utilizado para encerrar a produção dos dados, quando se identificou que o conjunto dos depoimentos era suficiente para apreender o objeto de estudo^(17)^.

### Coleta e organização dos dados

Os participantes foram selecionados por conveniência e abordados pessoalmente, de maneira individual, nos locais onde atuavam. Os que aceitaram foram conduzidos a uma sala previamente reservada com os gestores dos serviços, assegurando privacidade e um ambiente favorável ao diálogo, dispondo de álcool em gel 70% e estrutura para aumentar a segurança sanitária no contexto pandêmico da COVID-19. Nessa oportunidade, apresentaram-se aos participantes o projeto de pesquisa e o TCLE para esclarecimentos e assinatura, declarando formalmente o aceite.

A produção dos dados ocorreu no período de maio a agosto de 2021, por meio de entrevistas individuais, conduzidas somente pela primeira autora, enfermeira e discente do Programa de Residência Multiprofissional em Estratégia Saúde da Família de uma universidade pública sediada em Belém. Durante a entrevista, estiveram em sala apenas a pesquisadora e o participante, sendo que ela foi previamente capacitada em reuniões de orientação para atividades de pesquisa, compondo a carga horária teórica do curso de residência.

Considerando que os serviços da APS e o Departamento de Vigilância em Saúde do município foram campos de estágio da pesquisadora durante o curso, ela naturalmente estabeleceu prévia relação com uma parcela dos profissionais que neles atuavam. Em vista disso, todos os participantes conheciam a trajetória e certas aspirações acadêmicas e profissionais da pesquisadora, bem como o fato de que este estudo figurava como requisito obrigatório para concluir o curso.

Visando guiar as entrevistas, utilizou-se roteiro semiestruturado, elaborado pela equipe de pesquisa e constituído por duas seções. Com sete questões sobre o perfil sociodemográfico e profissional, a primeira seção permitiu registrar o cenário de atuação dos participantes, idade, sexo, categoria profissional, titulação, tempo de atuação no serviço e participação em espaços de educação permanente. Com nove questões subjetivas, a segunda possibilitou explorar o objeto de estudo, focando nos saberes acerca da violência contra mulheres negras e nas práticas assistenciais e gerenciais diante dessa condição, as quais foram desdobradas no diálogo com os participantes.

Não foi realizado teste-piloto ou estudo de validação com esse instrumento, assim como não se utilizou diário de campo ou outra técnica complementar para produção dos dados, pois as entrevistas se mostraram suficientes para apreender o objeto. Considerando a completude e clareza dos dados, não foi necessário repetir entrevistas, optando-se, também, por não compartilhar as transcrições com os participantes, para que pudessem conferi-las e referendá-las, visando não comprometer a espontaneidade dos depoimentos. Após consentimento, as entrevistas foram audiogravadas em formato MP3 com dispositivo eletrônico, e duraram, em média, 40 minutos.

### Análise dos dados

As entrevistas foram transcritas e reunidas em um *corpus*, atendendo às exigências do *software* de análise lexical *Interface de R pour les Analyses Multidimensionnelles de Textes et de Questionnaires* (IRaMuTeQ^*®*^), versão 0.7, *alpha* 2, com vistas ao seu processamento. O IRaMuTeQ^*®*^ utiliza funcionalidades do *software* R, possibilitando a análise estatística e textual do *corpus*, com confiabilidade e rigor técnico-científico^(18)^.

No processamento, o *software* gerou um dendrograma, representação gráfica da Classificação Hierárquica Descendente (CHD), uma das modalidades analíticas do IRaMuTeQ^*®*^. Com a CHD, o *corpus* foi desdobrado em classes lexicais, constituídas por palavras organizadas verticalmente, segundo seu qui-quadrado (X^2^), valores estatísticos que demonstram a força associativa das palavras para formar as classes. Os segmentos de texto (ST) correspondentes a cada palavra foram lidos atentamente para compreender e inter-relacionar seus significados, propiciando o agrupamento das classes em eixos temáticos^(19,20)^.

Ressalta-se que, ao processar a CHD, o IRaMuTeQ^*®*^ identifica a melhor possibilidade para formar classes de acordo com os fatores estatísticos e semânticos do *corpus*. Muitas vezes, isso determina o aproveitamento de uma parcela dos ST, considerando 75% como percentual mínimo para que a análise seja confiável^(19,20)^.

Assim, dois eixos derivaram da análise dos dados subjetivos, cada um constituído por três classes, segundo suas complementaridades e similaridades, os quais foram interpretados e discutidos com base na literatura científica pertinente sobre o tema e em documentos oficiais de cunho jurídico e da saúde. Por sua vez, os dados do perfil sociodemográfico e profissional foram organizados em planilha do *Microsoft Office Excel^®^*, versão 2016, e analisados com estatística descritiva para obter os valores absolutos e respectivos percentuais. Os processos de análise e interpretação dos dados foram conduzidos pelos cinco primeiros autores.

No intuito de preservar sua originalidade, o relatório do estudo não foi encaminhado aos participantes, motivo pelo qual não forneceram opiniões sobre os resultados, mas pretende-se compartilhar o artigo tão logo seja publicado.

## RESULTADOS

Dos 34 participantes, 28 (82,35%) atuavam em atividades assistenciais na APS, e seis (17,65%), como técnicos do Departamento de Vigilância em Saúde, sendo 24 (70,59%) do sexo feminino. A idade variou de 29 a 56 anos, com média de 35 e predomínio da faixa de 40 a 49 anos (n=12; 35,29%). No tocante à categoria profissional, 24 (70,59%) eram enfermeiros, cinco (14,71%) médicos, três (8,82%) fisioterapeutas e dois (5,88%) terapeutas ocupacionais. Quanto à titulação, 25 (73,53%) declararam ter pós-graduação *lato sensu* e/ou *stricto sensu*.

O tempo de atuação profissional foi assim distribuído: 15 (44,12%) referiram seis a dez anos, seguidos por oito (23,53%), com um a cinco anos; sete (20,59%), com 11 a 15 anos; três (8,82%), com 21 anos ou mais; e um (2,94%), com 16 a 20 anos. Quanto à educação permanente, 31 (91,18%) relataram nunca ter participado de espaços formativos sobre violência contra mulheres.

O *corpus* foi constituído por 34 textos, correspondendo ao conjunto de entrevistas. Identificaram-se 94 ST, com aproveitamento de 77, perfazendo 81,91% do *corpus*, motivo pelo qual este estudo revelou os principais dados subjetivos e que permitiram apreender o objeto de estudo, dispensando os dados secundários, constituintes do percentual não aproveitado do *corpus* (18,09%).

Emergiram 3.228 ocorrências (formas ou palavras), sendo 1.048 palavras distintas e 678 com única ocorrência (hápax). Por meio da CHD, o *corpus* subdividiu-se em três *subcorpora* e seis classes lexicais. Seguindo a ordem de partição, o primeiro *subcorpus* se constituiu pela classe 6, o segundo, pela classe 4, e o terceiro, pelas classes 5, 1, 2 e 3 (Figura 1).

**Figura 1:** Dendrograma da Classificação Hierárquica Descendente, Belém, Pará, Brasil, 2021

Formada por 10 ST (12,99% do *corpus*), a classe 6 apresenta 16 palavras representativas, considerando os valores de X^2^. Por sua vez, a classe 4 apresenta 18 ST (23,38%) e 17 palavras representativas, sendo a maior; a classe 5, 12 ST (15,58%) e 18 palavras representativas; a classe 1, 15 ST (19,48%) e 14 palavras representativas; a classe 2, 12 ST (15,58%) e 11 palavras representativas; e a classe 3, 10 ST (12,99%) e 13 palavras representativas. Assim, juntas, as classes 6 e 3 constituem as menores (Figura 1).

Organizando as classes em dois eixos temáticos, o primeiro eixo agrupou as classes 6, 3 e 2, e o segundo, as classes 4, 5 e 1. Esses eixos foram nomeados de acordo com o conteúdo das classes, e encontram-se a seguir, destacando palavras representativas, significados e alguns excertos emblemáticos.

### Eixo temático 1 - Saberes dos profissionais de saúde sobre a violência doméstica contra mulheres negras (classes 6, 3 e 2)

Este eixo apresenta o entendimento dos profissionais acerca da violência doméstica, explicando que o reconhecimento da violência ocorre tanto pelo conjunto de atos sutis quanto por ações danosas, visto que todos constituem práticas violentas e dominadoras contra mulheres negras. Essa compreensão abrange aspectos da segurança pública, que são entendidos como frágeis para combater a violência e suas possíveis repercussões na vida da mulher vitimada.

Palavras da classe 6, como “querer” (X^2^=20,91), “esposo” (X^2^=7,96) e “companheiro” (X^2^=7,89), revelam que os profissionais já detectaram algumas formas de violência, a exemplo da violência sexual, com o companheiro exercendo dominação sobre a mulher na prática de relação sexual não consentida. Além disso, outras práticas violentas provocam medo e dependência, sejam elas de cunho afetivo, financeiro e/ou psicológico. Na percepção dos profissionais, essas práticas resultam em danos às mulheres:

*O companheiro ou o esposo se acha no direito de mandar na mulher, e ela possui o dever de obedecer, sem ter escolha. Muitas relatam assim: “Às vezes, não quero ter relação sexual, mas fico muito submissa”. Elas são dependentes do marido, não correm atrás do que desejam realmente.* (PA 11)

*Esposo que violenta a mulher provoca situações de dependência, e, por medo, pela insegurança, pelo receio de ficar sozinha, por não ter como se manter, entre outras situações,* [ela] *acaba continuando* [com o marido]. (PA 14)

Quanto às mulheres negras, algumas palavras da classe 3, como “racismo” (X^2^=14,36) e “preconceito” (X^2^=5,11), mostram o reconhecimento de que, no caso delas, a violência é ainda mais grave, pois traz o componente racista, que constitui um forte legado na reprodução do pensamento coletivo ao longo do tempo:

*Violência contra a mulher é bater, é a humilhação, a manipulação* [psicológica]*, tudo que machuca. Mas o preconceito de cor é cruel. Infelizmente, existe racismo.* (TV 1)

*As mulheres negras sofrem muito mais. Ser negro é carregar um fardo.* (PA 5)

Na classe 2, destaca-se que a palavra “pandemia” (X^2^=3,80) reflete o contexto de crise sanitária provocado pela COVID-19 e por suas repercussões nos âmbitos social e da saúde, com aumento do número de casos de violência doméstica em virtude da convivência forçada/imposta pelas medidas sanitárias, principalmente nos períodos de *lockdown:*

*A violência aumentou muito nesse período de pandemia. Estamos vendo nos jornais muitos feminicídios, principalmente homens matando suas esposas, namoradas* [...]. (TV 6)

*O silêncio ficou mais forte na pandemia* [...]*, não pode ter contato para conversar* [devido às restrições para controlar a COVID-19]*. A mulher permanece em uma relação violenta por muitas razões: medo, vergonha, esperança de que o comportamento do parceiro mude, dependência econômica, psicológica e isolamento social.* (PA 13)

Nesse contexto, as palavras “respeito” (X^2^=14,36) e “amor” (X^2^=9,96), que figuram na classe 3, demonstram que os profissionais compreendem a violência, inclusive contra mulheres negras, como fenômeno motivado, sobretudo, pela dinâmica da relação interpessoal entre vítima e agressor. Isso retrata valores e normas sociais que podem influenciar o cuidado integral à vítima:

*Muitos feminicídios, principalmente homens matando suas esposas ou namoradas,* [ocorrem] *por não existir mais respeito e amor. Esse negócio de cor, para mim, não tem importância. Agora todo mundo fala “vidas negras importam”, mas temos que falar que todas as vidas importam, principalmente esses casais* [que vivem em conflito]*, para restabelecer o amor e diminuir o ódio.* (TV 6)

*Inconscientemente, todos nós julgamos alguém, mesmo não querendo. Tenho um carinho enorme pelos meus pacientes. Houve vezes que eu os julguei silenciosamente, e compreendo por tudo que eles passaram, mas acho que a permissão da falta de respeito na relação provoca a violência. O amor é necessário numa relação saudável.* (PA 28)

Os profissionais fortemente apontam que, em muitos contextos, a estrutura social ainda está centrada na figura masculina como detentora e influenciadora das relações amorosas, da mesma maneira que o pensamento social, quando ancorado no racismo, impulsiona o fenômeno da violência:

*Ainda nos deparamos com indivíduos que acreditam na superioridade do ser humano em razão da cor de sua pele e da mulher como objeto. A gente vê muitos casos de homens que não aceitam a separação, batem* [na mulher]*, quebram as coisas, ameaçam e matam, e ainda tem aqueles que escondem o corpo.* (TV 4)

*A violência doméstica, com certeza, dói mais na mulher negra, sendo que ela é a mais vulnerável, se levar em consideração fatores como desemprego e dependência emocional. Nessa família, normalmente é o homem quem trabalha* [...]. (PA 5)

Essa dimensão sociocultural, apresentada pelas palavras “homem” (X^2^=20,18) e “muito” (X^2^=11,11), na classe 2, expõe a lógica patriarcal e a influência capitalista que, juntas, determinam estereótipos para homens e mulheres no imaginário social e a dinâmica de uso do poder na violência doméstica. Ressalta-se que, ao discorrerem sobre o fenômeno, por vezes os profissionais generalizam o contexto feminino, sombreando aspectos que constituem o universo social das mulheres negras, mas que precisam ser reconhecidos em suas singularidades:

*Os homens que são agressores por natureza vão questionar suas vestimentas, sua conduta, o horário em que a mulher estava na rua; vão minimizar seu relato e questionar sua palavra. A sociedade é baseada nisso: o homem tem o poder! Até pouco tempo, eles que dominavam o mercado* [de trabalho]*. Mas as mulheres têm que ter cuidado e sempre driblar esse poder.* (PA 2)

*O homem, historicamente, acha que é dono da mulher. A cultura ainda é muito presente de o homem ser detentor de tudo, principalmente na relação* [amorosa]. (PA 4)

Por outro lado, as palavras “Brasil” (X^2^=20,91), “lei” (X^2^=7,96) e “infelizmente” (X^2^=7,96), na classe 6, caracterizam a violência doméstica, principalmente no cotidiano de mulheres negras, como problema social de proporção nacional, com elevado grau de injustiça. Os participantes expressaram fragilidades que ocorrem na aplicação oportuna e efetiva das medidas de proteção, previstas em lei, prevalecendo condições de impunidade, na medida em que a vítima, após recorrer às autoridades constituídas para esse fim, muitas vezes ainda estará sujeita ao risco de sofrer outros episódios de violência, inclusive de maneira letal:

*O Brasil é um país extremamente desigual, com uma legislação penal arcaica,* [o que] *resulta nisso* [violência doméstica]*. Ela* [mulher] *vai denunciar o companheiro, mas ele não vai ser preso. Quando ela morrer,* [as autoridades] *vão ver a denúncia.* (PA 15)

*A criminalidade e a impunidade, por causa das leis* [que não são cumpridas]*, são um grande problema, pelo menos no Brasil, e acredito que isso seja para todos, homens e mulheres.* (PA 18)

Nesse contexto, ressalta-se também a percepção de que a passividade feminina favorece a violência como um “atenuante”, pois o fato de a mulher não reagir constitui uma espécie de aceitação. Assim, o fenômeno é reduzido a expressões individualizadas sobre o relacionamento da vítima com o agressor. Contudo, essa representação da coletividade, que tenta explicar o ciclo da violência doméstica, não considera os marcadores sociais do fenômeno:

[...] *a maioria dos casos de violência doméstica contra a mulher vem de problemas com álcool, drogas ou doenças mentais do marido.* (TV 2)

*Violência é atacar a pessoa de qualquer maneira. O fato de ocorrer na relação amorosa, com a presença de sentimento,* [faz com que] *o amor intenso entre o casal reduza a dor do momento, e nasce um ciclo vicioso de abusos. O homem muda suas atitudes após a violência e oferece uma convivência agradável, que ameniza a situação. A mulher é tranquilizada com as atitudes românticas do marido. Notamos que o problema logo foi resolvido. Se a situação fosse tão grave, a mulher abandonaria logo o agressor/companheiro.* (PA 3)

### Eixo temático 2 - Práticas assistenciais e gerenciais diante da mulher negra em situação de violência doméstica (classes 4, 5 e 1)

Este eixo possibilita compreender a atuação dos profissionais diante da mulher negra em situação de violência doméstica no âmbito da APS.

As palavras identificadas na classe 4, como “notificação_compulsória” (X^2^=25,04), “denúncia” (X^2^=9,57) e “realizar” (X^2^=9,57), apontam para os desafios da notificação dos eventos de violência interpessoal na APS. As palavras estão associadas à ideia da notificação como elemento de prova criminal para a justiça civil, ainda que o registro da ficha de notificação sirva apenas para fins epidemiológicos, resguardando legalmente o profissional notificante:

*Eu acredito na notificação compulsória para além de fins epidemiológicos. Caso aconteça aqui, irei identificar na notificação, colocar no prontuário e aconselhar.* (PA 5)

*Muitas mulheres acreditam que podem suportar as agressões e continuar no relacionamento como forma de proteger os filhos. A notificação compulsória e qualquer outro tipo de denúncia não são realizados aqui, levando em consideração essas situações. É como o profissional de saúde se sujeitará à contradição, gerando a diminuição da sua credibilidade profissional.* (PA 26)

O entendimento dos profissionais sobre a notificação compulsória como um tipo de denúncia civil aponta, explicitamente, a insegurança ao realizá-la, pois consideram a violência doméstica como um ciclo que não será interrompido facilmente, sobretudo quando existe algum tipo de dependência da vítima em relação ao agressor.

Nesse sentido, palavras como “caso” (X^2^=23,81) e “polícia” (X^2^=17,53), junto às palavras da classe 5, como “acontecer” (X^2^=28,96) e “agressão” (X^2^=22,70), apresentam o contexto de negação das atribuições do setor saúde diante da violência contra mulheres negras, evidenciando o retrato do racismo institucional na saúde pública, o qual, muitas vezes, ocorre pelo medo que o profissional manifesta diante da possibilidade de também se enquadrar em situação de vulnerabilidade:

*Violência é caso de polícia. Não tem porquê estarmos aqui, na unidade de saúde, problematizando essa questão.* (PA 9)

[...] *acho que, dependendo da gravidade* [do caso]*, não farei o atendimento. Fico com receio, pois pode acontecer de aquela mulher voltar* [reatar] *com o marido depois da agressão, e eu, enfermeira, ficar marcada por ele* [...]*, a dor causada pela violência deve ser denunciada para polícia.* (PA 20)

Assim, consideraram que a violência deve ser tratada apenas no âmbito da segurança pública, como evidenciado pela insegurança dos profissionais para executar ações de cuidado à vítima, somada ao receio de retaliações do agressor ao profissional no próprio ambiente de trabalho e/ou em outros ambientes, inclusive pela possibilidade de se tornarem vítimas letais da violência. Esse retrato da negação e do pacto de silêncio na saúde pública, especialmente na APS, em um contexto pandêmico, possivelmente intensificou um cenário sombrio para o feminicídio.

Ao identificarem situações de violência doméstica, dependendo das características e da intensidade das lesões, aconselhavam as vítimas, encaminhando-as a outros níveis de atenção à saúde. Em vista disso, na classe 4, palavras como “ajudar” (X^2^=20,49) e “orientar” (X^2^=13,83) estão associadas às condutas de resolutividade ao deparar-se com uma mulher negra em situação de violência. Contudo, as práticas referidas denotam que os profissionais as reduziram aos cuidados para tratar agressões físicas leves, evidenciando limitações no cuidado ampliado. Reconheceram que é necessária a participação de uma equipe multiprofissional como suporte para as ações de cuidado, apontando profissionais que podem compô-la, como psicólogo e assistente social:

*Acho também que o que pode ajudar a fortalecer são os profissionais como psicólogo e assistente social, que* [ajudam a reduzir] *a dor da violência.* (PA 20)

[...] *hoje, na unidade* [de saúde]*, se ocorrer uma situação, com sinais evidentes de agressão, violência propriamente dita contra a mulher negra, irei apenas fazer cuidados de saúde, que não irão ampliar aos cuidados de cunho social.* (PA 24)

Por fim, as palavras “agressor” (X^2^=12,49) e “violência” (X^2^=2,24), na classe 1, junto às palavras “sofrer” (X^2^=6,19), “família” (X^2^=6,19) e “relação” (X^2^=2,42), na classe 5, engendram o reconhecimento acerca das repercussões da violência aos membros da família. No contexto intrafamiliar, a violência doméstica é encarada como fator agravante para mulheres negras, com efeitos nocivos aos seus filhos. Compreendendo que esse fenômeno representa um ataque indireto à prole, os profissionais declararam que situações de violência implicam danos sociais e de saúde também para os filhos, demandando outras ações de cuidado:

*Quando a agressão acontece com a mulher, ela é danosa por si,* [pois] *causa danos em toda a estrutura da família, principalmente quando existem filhos. Nesse caso,* [também] *é violência para os filhos.* (PA 1)

*A violência causa dor nas mulheres, e todos à sua volta sofrem.* (PA 21)

Constatou-se que os profissionais exploraram fortemente a ideia de dependência das vítimas que têm filhos, pois foram encaradas como as que mais suportam situações de agressão, no intuito de protegê-los e evitar que sofram privações e/ou abalos psicológicos/emocionais.

## DISCUSSÃO

Os dados evidenciaram os saberes dos profissionais acerca da violência doméstica contra mulheres negras e um conjunto de práticas diante dessa realidade. Sabe-se que essa violência engloba a interseção complexa das opressões sociais, contexto ampliado e que pode ser didaticamente entendido como um intercruzamento dos âmbitos microssocial e macrossocial. O âmbito microssocial corresponde às representações e ações singulares dos sujeitos, diferenciando-se do macrossocial, apontado pelas implicações estruturais de extensão histórica, política e social, que envolvem a sociedade e, consequentemente, repercutem nas mulheres^(21)^.

No contexto da violência, o reconhecimento do âmbito microssocial é marcado pelas relações de poder entre homens e mulheres, enraizadas pelo sistema patriarcal, no qual a dominação nas relações de gênero influenciam as práticas cotidianas e tem como alicerce o engessamento da definição de papéis sociais. Essas formas de dominação e exploração se relacionam, de maneira recíproca, em uma estrutura composta por classe, gênero e raça^(22-24)^.

As expressões de violência contra mulheres negras encontram convergências e possibilidades de interpretação à luz de referenciais teóricos disponíveis na literatura científica, como a teoria da interseccionalidade, por meio da qual se analisa como determinadas categorias sociais e culturais se entrelaçam, abrangendo relações entre vários elementos, destacando-se etnia e sexualidade, mas também classe, gênero e raça, em uma perspectiva multidimensional^(21)^.

Faz-se tal ponderação considerando que essa teoria permite refletir sobre o fato de muitas formas de opressão e suas iniquidades correspondentes estarem firmemente entrelaçadas e reforçarem-se mutuamente, criando experiências únicas de discriminação. Isso tende a gerar como produto a maior vulnerabilidade do público feminino à violência, tanto no ambiente doméstico quanto em outros espaços sociais, causando ou exacerbando fenômenos de marginalização, sobretudo entre mulheres negras^(21)^.

Assim, os resultados desta pesquisa vão ao encontro de estudo realizado em Marabá (PA), que analisou as percepções de profissionais de saúde sobre o tema, indicando o ambiente doméstico como epicentro da violência contra mulheres, bem como o íntimo vínculo domiciliar com o agressor e o medo de romper a situação de violência como seus principais influenciadores^(25)^. No mesmo estudo, constatou-se que, por vezes, o desrespeito à mulher extrapola o ambiente domiciliar e alcança o ambiente jurídico, caracterizando-se, no entanto, por obstáculos de proteção à mulher e pelo julgamento ineficaz do agressor^(25)^.

Embora os resultados desta pesquisa apontem para a violência contra mulheres negras, cometida por parceiros íntimos no ambiente doméstico, a violência se estende a uma pluralidade de agressores, podendo ser compartilhada por grupos familiares e outros grupos sociais. Além disso, pode abranger diferentes padrões de interação, fundamentando-se no racismo e/ou sexismo. Essa diversidade deve ser considerada para ampliar o escopo de enfrentamento mediante o reconhecimento das desigualdades sociais existentes^(26)^.

Como violação de direitos humanos, a violência doméstica produz efeitos diretos à saúde, podendo ser letal às vítimas. Nesse sentido, os participantes reconheceram a violência para além dos aspectos físicos, incluindo suas repercussões psicossociais. No entanto, manifestavam insegurança quanto às ações que deveriam empreender, expondo a necessidade de aprimorar habilidades assistenciais/gerenciais e qualificar a segurança pública para que tenham a segurança necessária para acolher as vítimas, solucionar a condição de violência e/ou mitigar as suas repercussões biopsicossociais^(25)^. Isso pode ocorrer sobretudo na APS, cujo relacionamento aproximado entre profissionais e grupos humanos possibilita enfrentar diferentes agravos^(27-29)^.

Concepções populares arcaicas e equivocadas, como a ideia de que mulheres “gostam de apanhar”, negam a complexidade do problema, de modo que a permanência de vítimas com agressores é banalizada e não encarada como resultado de múltiplos fatores, atribuindo à violência um caráter individual oriundo de aspectos supostamente específicos da personalidade feminina, como o nível de tolerância aumentada e/ou a esperança na transformação do agressor. Essas concepções resultam na negação do ciclo de violência e no silenciamento das vítimas^(27)^.

Assim, é imprescindível analisar os fatores sociais conjuntamente por classe, gênero e raça. Pautada na interseccionalidade, essa análise possibilita compreender as formas como operam o racismo, o patriarcalismo, a opressão de classe e outros sistemas discriminatórios, que criam desigualdades e estruturam/institucionalizam a subordinação e as vulnerabilidades entre pessoas^(23,30)^.

Diante da multiplicidade do gênero feminino, identifica-se a união dos fenômenos racismo e sexismo, elementos que produzem efeitos violentos sobre a mulher, principalmente a mulher negra^(31)^. Essa lógica opressora foi reproduzida nos achados deste estudo, quando apontaram certos saberes dos profissionais, ancorados na relativização do sofrimento provocado pela violência, associando-a com aspectos afetivos e de foro íntimo como resultado da expressão de subjetividade social do racismo e sexismo, da qual, muitas vezes, nem eles se dão conta.

As ações de enfrentamento da violência foram amplamente impactadas pela pandemia de COVID-19, que modificou o acesso aos serviços da APS para reduzir a propagação do vírus, bem como a dinâmica relacional das famílias pela necessidade de seus membros permanecerem isolados no ambiente doméstico por dias ou semanas, aumentando o número de casos de violência contra mulheres. Nesse cenário, evidências apontam que elas foram as mais afetadas pela violência durante e após o confinamento^(32-34)^.

Situações caracterizadas pelo aumento do estresse devido ao fechamento de escolas e altas taxas de desemprego, além da necessidade de algumas mulheres permanecerem em confinamento no mesmo domicílio que seus agressores, aliadas à dependência financeira em relação a eles, entre outros fatores ampliados pela pandemia, agravaram o cenário de violência doméstica. Além disso, a subnotificação de eventos violentos e o isolamento social das vítimas, resultantes da pandemia e da carência ou falta de redes de apoio, contribuíram para fortalecer a violência doméstica^(34)^.

Nesse contexto, os desafios cotidianos da rede de atenção à violência foram perpassados por fatores como a reinvenção das formas de criar e recriar vínculos com a comunidade, com restrições orçamentárias na APS e incorporação de normativas de cunho neoliberal que, por sua vez, resultaram na priorização dos atendimentos às demandas agudas, de origem biológica, em detrimento do cuidado integral e longitudinal, com abordagem comunitária e familiar^(35)^.

Portanto, na perspectiva da interseccionalidade, entende-se que é necessário reconhecer a violência contra mulheres como uma pandemia silenciosa e invisível, construída como fenômeno social e processada continuamente nas sombras das relações entre os grupos humanos, como ocorreu em meio à COVID-19^(21)^. Isso aponta a necessidade de promover ações intersetoriais direcionadas ao público feminino, sobretudo às mulheres negras, no intuito de garantir vínculos com os serviços, proteção e cuidado integral, considerando suas particularidades^(34)^.

As dificuldades no cuidado integral e os impasses da notificação compulsória, mencionados pelos profissionais, demandam o aperfeiçoamento da estrutura técnico-operacional da APS, com a implementação de protocolos e fluxos de atendimento para as atividades de prevenção, acolhimento/atendimento, notificação e seguimento dos casos, bem como para as atividades de promoção da cultura da paz e monitoramento epidemiológico da violência doméstica^(36,37)^.

Ressalta-se a importância de fortalecer a articulação dessas atividades com os demais níveis de atenção à saúde, conforme as instruções da Lei Orgânica da Saúde nº 8.080/1990^(38)^, incorporando a área da segurança pública, como orienta a Lei nº 14.550/2023, que trata da implementação de medidas protetivas de urgência às vítimas e aos seus dependentes^(39)^.

No tocante ao amparo legal, as Leis nº 10.778/2003 e nº 13.931/2019 tratam da identificação e notificação compulsória de casos suspeitos e confirmados de violência contra mulheres. Definem, em conjunto, as medidas que devem ser realizadas nos setores público e privado, destacando-se, no campo da saúde, o acolhimento e a notificação como objetos de identificação para providências cabíveis e fins estatísticos. Além disso, apontam a obrigatoriedade de comunicar a ocorrência à autoridade policial nas primeiras 24 horas^(40,41)^.

Diante da necessidade de notificar a violência, enfrenta-se a dificuldade de manejar situações complexas que a envolvem, como o medo de torna-se vulnerável diante do ato de notificar e realizar denúncia policial, expondo ideias que remetem à necessidade de delegar a outros setores a responsabilidade pela execução desse procedimento, como setores da área cível/criminal^(36,37)^.

Os desafios de notificar casos de violência doméstica e lidar com eles podem estar relacionados à falta ou carência de formação qualificada^(42)^, identificando-se os sentimentos de impotência e despreparo perante as situações vivenciadas na prática, os quais revelam falhas no acolhimento às vítimas. Portanto, é imprescindível promover atividades de educação permanente, no intuito de garantir bom atendimento à mulher vitimada, com base na equidade, integralidade e universalidade, princípios doutrinários do SUS^(43)^.

### Limitações do estudo

Como limitação, pontua-se que o estudo foi realizado em um município do Pará, mas entende-se que esse aspecto tem representatividade, por conta dos importantes indicadores de violência nesse cenário. Visando ampliar o escopo de análise, é necessário incorporar outros municípios, com representatividade das regiões de saúde do estado, possibilitando articular a rede de atenção à mulher negra em situação de violência tanto na saúde quanto em outros setores institucionais.

Além disso, os depoimentos sobre a complexa realidade que envolve a violência foram impactados pela pandemia de COVID-19, que interferiu na disponibilidade e no dimensionamento de recursos humanos, bem como na dinâmica dos serviços da APS e da Vigilância em Saúde.

### Contribuições para as áreas da enfermagem, saúde ou políticas públicas

Entende-se que o estudo contribui com a análise crítica de fatores que repercutem no enfrentamento da violência doméstica, sobretudo no cotidiano de mulheres negras, servindo de aporte teórico para auxiliar tanto as autoridades públicas, a discutir, implementar e avaliar medidas que solucionem esse problema ou minimizem suas repercussões, quanto os profissionais de saúde, a refletir sobre o tema e repensar suas ações diante dele.

## CONSIDERAÇÕES FINAIS

Evidenciou-se que os saberes dos profissionais sobre a violência contra mulheres negras se pautaram no reconhecimento da relação desigual de poder entre os gêneros, caracterizando-se por condições de subordinação e vulnerabilidade. Nas suas experiências, a violência doméstica praticada por cônjuge como principal agressor foi identificada como situação predominante.

Todavia, constataram-se limitações nas condutas de enfrentamento que podem contribuir para reforçar a vulnerabilidade das vítimas. Essas limitações devem ser reconhecidas para garantir bom atendimento nos serviços de saúde, com base na equidade, integralidade e universalidade.

Assim, é fundamental que as atividades da APS e da Vigilância em Saúde sejam contempladas com estrutura e instrumentalização técnico-operacionais adequadas, aproximando-as de outros setores estratégicos, a exemplo da segurança pública, no intuito de estabelecer o cuidado integral às mulheres que vivenciam esse grave problema.

## Data Availability

Os dados da pesquisa estão disponíveis no corpo do artigo.
